# Supplementary material for: Toxicity of Orthodontic Brackets Examined by Single Cell Tracking
Source: Toxics. 2022 Aug 8;10(8):460. doi: 10.3390/toxics10080460 (PMC9413677; doi:10.3390/toxics10080460)
Supplement: Supplementary file 1 [file toxics-10-00460-s001.zip › Supplementary Figures.pdf]

Supplementary Figures for:

Toxicity of Orthodontic Brackets Examined by Single Cell Tracking

Morgan Wishney [1], Swarna Mahadevan [2], James Anthony Cornwell [2,3], Tom Savage [4], Nick Proschogo [5], M Ali Darendeliler [1] and Hans Zoellner [2,6,7,8,*]

[1] Discipline of Orthodontics, Sydney Dental School, Faculty of Medicine and Health, University of Sydney, Sydney Dental Hospital, Surry Hills, NSW 2010, Australia

[2] The Cellular and Molecular Pathology Research Unit, Oral Pathology and Oral Medicine, Sydney Dental School, Faculty of Medicine and Health, The University of Sydney, Westmead Centre for Oral Health, Westmead Hospital, NSW 2145, Australia

[3] Laboratory of Cancer Biology and Genetics, Center for Cancer Research, National Cancer Institute,
Bethesda, MD 20892, USA

[4] School of Geosciences, Faculty of Science, The University of Sydney, Camperdown, NSW 2006, Australia

[5] School of Chemistry, Faculty of Science, The University of Sydney, Camperdown, NSW 2006, Australia

[6] Biomedical Engineering, Faculty of Engineering, The University of Sydney, Camperdown, NSW 2006, Australia

[7] Graduate School of Biomedical Engineering, University of NSW, Kensington, NSW 2052, Australia

[8] Strongarch Pty Ltd., Pennant Hills, NSW 2120, Australia

* Correspondence: hans.zoellner@strongarch.com; Tel.: +61-466-400-028

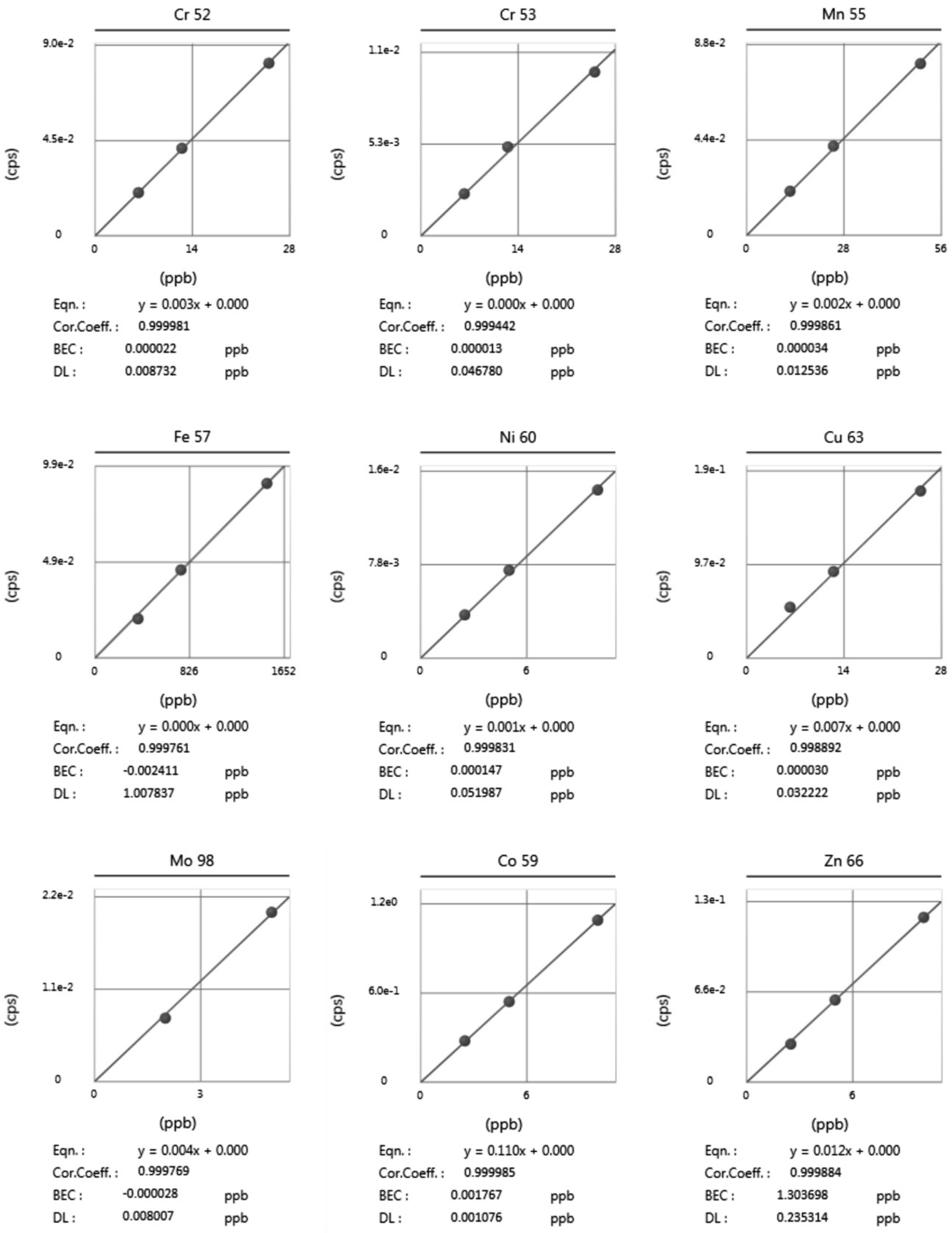

Figure S1. Graphs of ICP-MS standard curves for dilutions of commercially sourced standards for ^{52}Cr, ^{53}Cr, ^{55}Mn, ^{57}Fe, ^{60}Ni, ^{63}Cu, ^{98}Mo, ^{59}Co and ^{66}Zn, relating counts per second (CPS) to parts per billion (ppb). Linear relationships between the standard concentrations expected from dilutions and integrated ICPMS peaks were seen for all isotopes studied, and equations (Eqn.) for these are shown together with correlation coefficients (Cor. Coeff.). Calculated background equivalent concentrations (BEC) and detection limits (DL) for each calibrated isotope are shown.

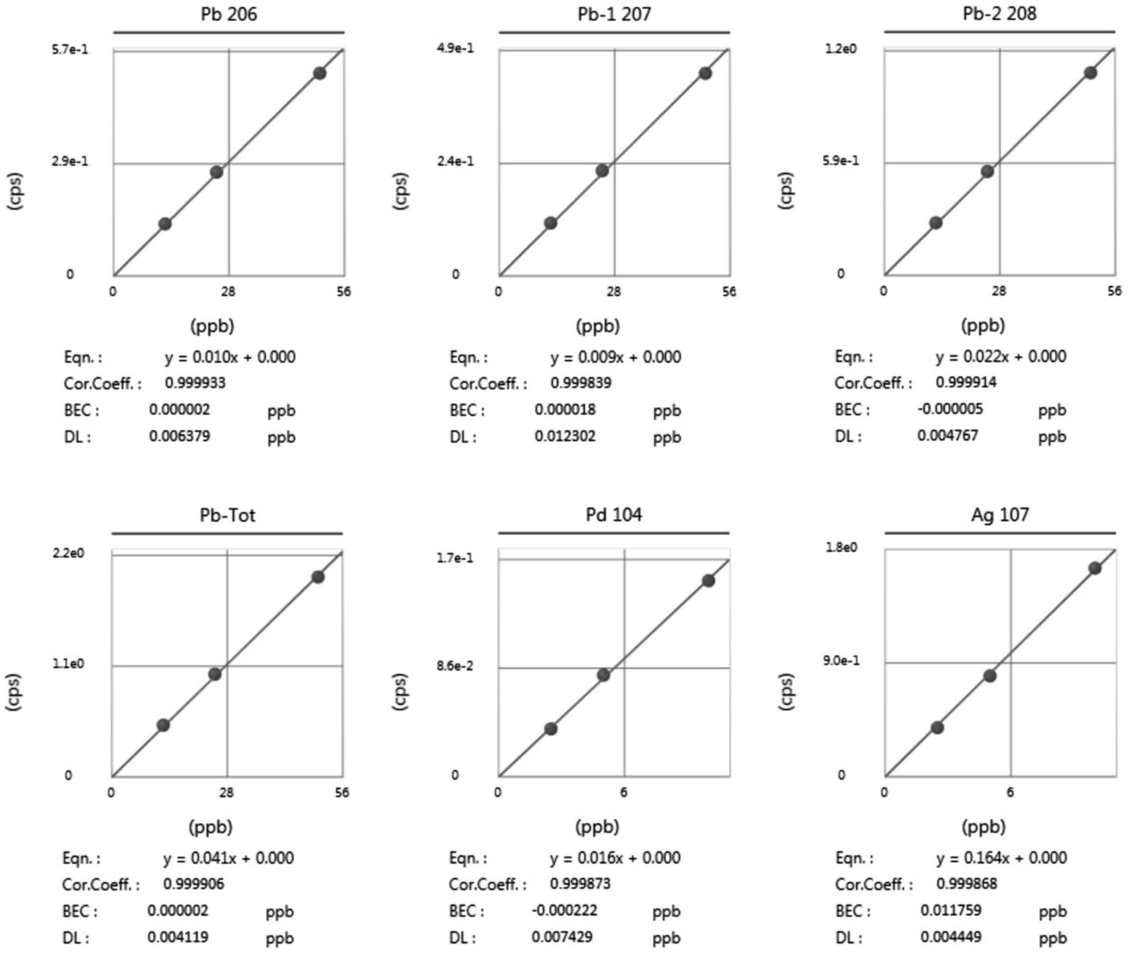

Figure S2. Graphs of ICP-MS standard curves for dilutions of commercially sourced standards for ^{206}Pb, ^{207}Pb, ^{208}Pb, ^{104}Pd and ^{107}Ag, as well as calculated summated Pb for all Pb isotopes (Pb-Tot), relating counts per second (CPS) to parts per billion (ppb). Linear relationships between the standard concentrations expected from dilutions and integrated ICPMS peaks were seen for all isotopes studied, and equations (Eqn.) for these are shown together with correlation coefficients (Cor. Coeff.). Calculated background equivalent concentrations (BEC) and detection limits (DL) for each calibrated isotope are shown.
